# Incorporating high-frequency information into edge convolution for link prediction in complex networks

**DOI:** 10.1038/s41598-024-56144-9

**Published:** 2024-03-05

**Authors:** Zhiwei Zhang, Haifeng Xu, Guangliang Zhu

**Affiliations:** School of Informatics and Engineering, Suzhou University, Suzhou, 234000 China

**Keywords:** Computer science, Information technology, Scientific data

## Abstract

Link prediction in complex networks aims to mine hidden or to-be-generated links between network nodes, which plays a significant role in fields such as the cold start of recommendation systems, knowledge graph completion and biomedical experiments. The existing link prediction models based on graph neural networks, such as graph convolution neural networks, often only learn the low-frequency information reflecting the common characteristics of nodes while ignoring the high-frequency information reflecting the differences between nodes when learning node representation, which makes the corresponding link prediction models show over smoothness and poor performance. Focusing on links in complex networks, this paper proposes an edge convolutional graph neural network *EdgeConvHiF* that fuses high-frequency node information to achieve the representation learning of links so that link prediction can be realized by implementing the classification of links. *EdgeConvHiF* can also be employed as a baseline, and extensive experiments on real-world benchmarks validate that *EdgeConvHiF* not only has high stability but also has more advantages than the existing representative baselines.

## Introduction

Numerous systems in nature and society can be characterized as complex networks^1–3^, such as World Wide Web, social networks, and biological networks, in which nodes represent entities and the connections between them are represented as edges or links (In this article, complex networks are depicted as graphs, with the terms ‘edge’ and ‘link’ being used interchangeably to signify the connections or relationships between nodes within the network. Furthermore, the terms ‘graph’ and ‘network’ both represent the same structural concept and are not differentiated in the context of this paper). Unfortunately, due to noise disturbance during graph data collection, we often lose several links between some nodes. In addition, complex networks themselves often evolve dynamically over time, and new links are often connected between some nodes. Fortunately, the technique of link prediction in complex networks aims to discover hidden or future links between network nodes, including the prediction of unobserved links, i.e., links that actually exist in a network but have not yet been detected, and the prediction of future links , i.e., links that do not exist in the network at present but should exist or are likely to exist in the future^3–9^. Link prediction, serving as an abstraction for numerous widespread issues, can be utilized in any system that transforms entities and their relationships into a network representation. This approach can enhance the effectiveness of biomedical experiments and can also be employed for completing knowledge graphs^2,3,10^.

In the biomedical field, whether there is a link between proteins needs to be inferred through a large number of expensive experiments. Taking the protein interaction network as an example, 80% of yeast protein interactions are still unknown, while only 0.3% of human interactions are known. However, if we design an accurate link prediction model based on the known network structure in advance, the predicted results can better improve the success rate of these experiments and reduce the experimental cost^10–13^. Link prediction also has an important application value for social network reorganization and structure function optimization. For example, based on the idea and method of link prediction, the category of unlabeled user nodes is predicted in the network where some node categories are known to judge whether a mobile phone user will change the communication operator. Citation networks, which are variants of social networks, are composed of references and cited relationships between literatures, contain research achievements in multiple fields and represent a considerable knowledge treasure in academia. Through link prediction techniques, researchers can easily obtain academic achievements that are most relevant to their own research content and closely track the latest scientific development trends^13,14^.

Graph neural networks (GNNs) implement graph deep learning technology, effectively learning node representations from complex networks and enhancing the performance of GNN-based link prediction models. Node representations in complex networks typically contain both low-frequency information, which represents shared node characteristics, and high-frequency information, which highlights node differences. However, existing GNNs tend to focus on low-frequency information, such as in the case of the representative graph convolutional network. By neglecting the high-frequency information in node representation, these GNNs produce similar or even indistinguishable node representations as the model’s depth increases, leading to a decrease in the performance of corresponding graph mining tasks.

To address the aforementioned issues, Shi et al. proposed fusing high-frequency and low-frequency information in node representation^15^ and obtained good model performance. Inspired by Shi et al.^15^ and based on our previous work^3^, this paper integrates the high-frequency information in the node representation of complex networks into the edge convolution graph neural network we have previously proposed, which focuses on the learning of link representation in complex networks rather than the learning of node representation, indirectly realizing link prediction through link binary classification.

Thus, two main contributions of this paper are as follows:

- We propose a complex network edge convolution operation by fusing high-frequency information in node representation, and construct an Edge Convolutional Network with High Frequency Information (*EdgeConvHiF*) for link prediction in complex networks.
- When building the *EdgeConvHiF* model, the normalization strategy of link representation is also introduced, which can better enhance the stability of the model.

This paper is structured as follows: section “Related works” presents an overview of the literature related to the topic under consideration. Section “Edge convolution based link prediction framework” describes the edge convolution-based approach to link prediction, which includes the fundamental concepts, edge convolution, extraction and fusion of high- and low-frequency information, and the construction of the link prediction model. In section “Experiments and discussion”, the experiments conducted to validate the proposed approach are presented and analyzed. Finally, the concluding remarks and potential avenues for future research are discussed in the last section.

## Related works

The core of GNN-based link prediction models lies in the construction and training of GNNs. Thus, in this section we will cover graph representation learning and GNN construction related to the issue of link prediction.

### Representation learning based link prediction

The primary objective of graph representation learning is to preserve the maximum amount of topological information when converting network nodes into vector representations. Graph representation learning is mainly divided into structure-based representation learning and feature-based representation learning. Structure-based representation learning only comes from the graph topological structure, that is, the graph structure represented by a two-dimensional adjacency matrix. However, feature-based representation learning contains both the topological and the corresponding feature information, such as the category of nodes and clustering coefficients.

Structure-based graph representation learning defines two structurally similar nodes in a graph as proximity, and our goal is to expect the learned node representation vector to be near in the vector space when it is approaching the graph. The DeepWalk random walk algorithm, introduced by Bryan and his team^16^, stands out as a key method in graph representation learning. The fundamental concept is to project the nodes’ relationships and structural characteristics into a new vector space, where nodes that are proximate in the graph also have closer proximity in the transformed vector space. Thus the graph data are converted into data in a vector space through such optimization goals, which lays a good foundation for the subsequent graph mining tasks, such as link prediction. Grover and his colleagues^17^ developed node2vec by generalizing DeepWalk in a wider context. This approach emphasizes community structure and node importance information, respectively. However, the LINE proposed by Tang et al. intuitively does not employ the random walk strategy^18^, but both LINE and DeepWalk apply the probability loss function, that is, minimization of the empirical probability of node connections and the node similarity distance after vectorization, and consider the first- and second-order similarity, which is similar to the internal motivation of the random walk strategy. Given that the aforementioned structure-based graph representation learning solely derives node representations from the graph topology, neglecting the nodes’ inherent attributes, the link prediction performance in certain intricate networks, particularly in social networks, is poor.

Fortunately, because the feature matrix of nodes is added to feature-based graph representation learning, the GNNs can more accurately obtain the representation of nodes so as to providing better support for the downstream tasks of graph mining. Thomas Kipf et al. proposed a Graph Convolutional Neural Network(GCN), the most representative feature-based GNN, to perform the semisupervised classification task of nodes in graph structured data^19^, which is modeled as a first-order approximation of spectral convolution and performs parameterized message passing operations in graphs. However, the GCN is essentially a low-pass filter so that it cannot effectively learn the high-frequency information in the graph. Then, William et al. presented an inductive framework GraphSAGE^20^ that leverages node attribute information to efficiently generate representations on previously unobserved data, which is characterized by a fixed sampling rate and different aggregation methods compared with the GCN, rather than a single hard aggregation neighbor node representation of the GCN. Better yet, Bengio et al. proposed GAT^21^, which combines adjacent nodes using the attention mechanism to dynamically assign varying weights to different neighbors, thereby significantly enhancing the representational capacity of the GNN model. In a nutshell, prevailing graph neural networks typically focus on learning low-frequency information from network nodes, while the acquisition of high-frequency graph data still requires further reinforcement.

### Graph neural network based methods

In contrast to conventional link prediction approaches, GNN-based link prediction initially utilizes a graph neural network to learn node representations, followed by performing relevant operations on the representations of a node pair, such as the Hadamard product, to yield specific outcomes. Subsequently, a classifier is applied to these results to ascertain the presence of a link between the given node pair. Kumar et al. have offered an extensive review of link prediction techniques, their applications, and performance, allowing readers to obtain more in-depth information from the cited literature^22^. Moreover, in our previous work on GNN-based link prediction for complex networks, we concentrated on link representation learning and developed an edge convolution operation to facilitate link representation learning^3^. We have also incorporated a normalization strategy for the learned link representation in order to improve the model stability within the edge convolution-based link prediction model. This is achieved by constructing the link prediction graph neural network *EdgeConvNorm* using a series of stacked edge convolution operations. Regrettably, *EdgeConvNorm* also falls short in learning and utilizing high-frequency information in network node representation. To address this shortcoming, we aim to further enhance *EdgeConvNorm * by incorporating both high-frequency and low-frequency data into the link prediction edge convolution operations, specifically, the *EdgeConvHiF* proposed in this paper.

## Edge convolution based link prediction framework

In this section, we initially present the relevant background information on link prediction. Subsequently, we will derive the edge convolutional operations incorporating high-frequency graph information in a step-by-step manner. Lastly, we provide an overview of the link prediction framework based on the edge convolution introduced in this paper.

### Preliminaries

#### Notations and symbols

To describe and explain the link prediction-related issues more clearly, the notations and symbols employed in this paper are listed in Table 1.

**Table 1:** Notations and symbols and their illustration employed in this paper.

Notations and symbols	Illustration
$\mathscr{G}=(\mathscr{V},\mathscr{E})$	${\mathscr {G}}$ indicates a graph, and ${\mathscr {V}}$ and ${\mathscr {E}}$ represent the node set and edge set of ${\mathscr {G}}$, respectively
${\mathscr {A}}$	${\mathscr {A}}$ represents the adjacent matrix of ${\mathscr {G}}$
*n* = $|\mathscr {V}|$	The amount of nodes in ${\mathscr {G}}$
$I_n$	An identity matrix with *n* elements
$D^{n\times n}$	The degree matrix of ${\mathscr {G}}$ with $n\times n$ elements, and $D_{ij}=\sum _{j=0}^{n-1}{\mathscr {A}}_{ij}$
E = $|\mathscr {E}|$	The amount of edges in ${\mathscr {G}}$
$\mathscr{V}=\{v_1,v_2,\ldots ,v_n\}$	$v_i$ depicts the *i*-th node in ${\mathscr {G}}$
$\mathscr {E}=\{e_1,e_2,\ldots ,e_n\}\subseteq \mathscr{V}\times \mathscr{V}$	$e_i$ represents the *i*-th edge in ${\mathscr {G}}$
${\mathscr {X}}=\{x_1,x_2,\ldots , x_n\}$	${\mathscr {X}}$ indicates the representation matrix of ${\mathscr {G}}$, while $x_i$ illustrates the representation of the *i*-th node in ${\mathscr {G}}$
${\mathscr {X}}_{lo}, {\mathscr {X}}_{hi}$	${\mathscr {X}}_{lo}$ and ${\mathscr {X}}_{hi}$ represent the low-frequency and high-frequency information, respectively
$e_{ij}$	The link between node $v_i$ and $v_j$
${\mathscr {N}}(i)$	The neighbors of node $v_i$
$\mathscr{U}=\mathscr{V}\times \mathscr{V}$	${\mathscr {U}}$ represents the universal edge set consisting of $n*(n-1)/2$ in undirected graphs or $n*(n-1)$ in directed graphs
${\mathbb {E}}={\mathscr {U}}-{\mathscr {E}}$	${\mathbb {E}}$ represents the unobserved links in ${\mathscr {G}}$
$h_i$	$h_i$ represents the embedding or representation of node *i*
$h_{i}^{l}$	$h_{i}^{l}$ represents the embedding or representation of node *i* in the *l*-th layer of a GNN
$h_{i}^{hi}, h_{i}^{lo}$	$h_{i}^{hi}$ and $h_{i}^{lo}$ represent the high- and low-frequency information of node *i*, respectively

#### Link prediction

Given a graph $\mathscr{G}=(\mathscr{V},\mathscr{E})$ with node set ${\mathscr {V}}$, observed link set ${\mathscr {E}}$ and the corresponding universal link set ${\mathscr {U}}$, link prediction predicts whether there is a link between two nodes $v_i$ and $v_j$ ($v_i, v_j\subseteq {\mathscr {V}}$) according to the known structure and attributes of ${\mathscr {G}}$. Formally, GNN-based link prediction can be illustrated by the following procedure. First, the observed link set ${\mathscr {E}}$ is divided into the training set ${\mathscr {E}}^T$ and validation set ${\mathscr {E}}^P$, while ${\mathbb {E}}$ serve as test dataset (Intuitively, based on specific requirements, the test set can also be assembled by selecting a designated number of edges at random from ${\mathscr {U}}$) to evaluate the link prediction model performance. Obviously, ${\mathscr {E}}^T \cap {\mathscr {E}}^P=\varnothing$, ${\mathscr {E}}^T \cup {\mathscr {E}}^P={\mathscr {E}}$, and ${\mathbb {E}}={\mathscr {U}}-{\mathscr {E}}$. Then, a GNN model ${\mathscr {M}}$ learns on ${\mathscr {E}}^T$ and validates on ${\mathscr {E}}^P$ to accomplish the training task. Finally, the corresponding link prediction performance evaluation measures, such as AUC (Area Under the Receiver Operating Characteristic Curve), are applied to the learned model ${\mathscr {M}}$ on the test dataset for performance evaluation.

### Edge convolution with high frequency information

To our knowledge and based on prior research, the edge convolution model *EdgeConv* was initially introduced by Wang et al.^23^ and was used for point cloud learning, as depicted in Eq. 1.

1 $$\begin{aligned} h_{i}^{(l+1)}=\max _{j\in {\mathscr {N}}(i)}{ReLU(\Theta \cdot (h_{j}^{(l)}-h_{i}^{(l)})+\Phi \cdot h_{i}^{(l)})} \end{aligned}$$

where both $\Theta$ and $\Phi$ are linear layers in *EdgeConv*. Although *EdgeConv* has achieved excellent performance on the point cloud of dynamic graphs with relatively dense structures in Euler space, its performance is not ideal in networks with relatively sparse structures, especially in social networks with strong sparsity. As illustrated in^23^, *EdgeConv*, in multi-layer systems, effectively captures semantic attributes across potentially extensive distances in the original embedding, while also accurately preserving the point cloud’s topological structure. Thus, inspired by Wang et al.^23^, we have made corresponding improvements to *EdgeConv* to make it better adapt to networks such as the citation network in^3^. The corresponding improvements to the edge convolution of *EdgeConv* are shown in Eq. 2.

2 $$\begin{aligned} h_{i}^{(l+1)}=\mathop {mean}\limits _{j\in {\mathscr {N}}(i)}{LeakyReLU(\Theta \cdot h_{j}^{(l)}+\Phi \cdot h_{i}^{(l)})} \end{aligned}$$

For computing manipulation and simple purposes, we further deduce Eq. 2 as Eq. 3.

3 $$\begin{aligned} h_{i}^{(l+1)}=\mathop {mean}\limits _{j\in {\mathscr {N}}(i)}{LeakyReLU(\Theta \cdot (h_{j}^{(l)}~||~h_{i}^{(l)}))} \end{aligned}$$

where || represents the concatenate manipulation of node representation. Thus, the representation of edge $e_{ij}$ can be learned from Eq. 4.

4 $$\begin{aligned} h_{e_{ij}}^{(l+1)}=(h_{i}^{(l+1)}~||~h_{j}^{(l+1)}) \end{aligned}$$

The explanation and deduction of Eqs. 2–4 were detailed in our previous work^3^, and the complete *EdgeConvNorm* model for link prediction based on edge convolution is available for readers to acquire.

Regrettably, in the process of learning link representation, both *EdgeConv* and *EdgeConvNorm* exclusively focus on low-frequency information, which captures the shared attributes of nodes, while neglecting the high-frequency information that highlights node differences. Consequently, it is a natural idea to incorporate both high- and low-frequency information in node representation, which can improve node representation learning and ultimately lead to better performance for the link prediction model.

Motivated by the idea of beyond low-frequency information in GCNs presented by Shi et al.^15^, this paper builds upon and refines the approach for extracting and integrating high- and low-frequency information in node representation.

#### Extraction of high- and low-frequency information

We simply employ the high-pass and low-pass filters proposed by Shi et al.^15^ to accomplish the extraction of high- and low-frequency information in node representation. Correspondingly, the low-pass filter ${\mathscr {F}}_{lo}$ and high-pass filter ${\mathscr {F}}_{hi}$^15^ are shown in Eqs. 5 and 6, respectively.

5 $$\begin{aligned} {\mathscr {F}}_{lo}= & {} \alpha I_n + D^{-1/2}AD^{-1/2} \end{aligned}$$

6 $$\begin{aligned} {\mathscr {F}}_{hi}= & {} \alpha I_n - D^{-1/2}AD^{-1/2} \end{aligned}$$

where $\alpha$ is a hyperparameter. Consequently, the low-frequency information ${\mathscr {X}}_{lo}$ and high-frequency information ${\mathscr {X}}_{hi}$ of ${\mathscr {G}}$^15^ can be obtained by Eqs. 7 and 8, respectively.

7 $$\begin{aligned} {\mathscr {X}}_{lo}= & {} ({\mathscr {F}}_{lo}\star {\mathscr {X}})_{{\mathscr {G}}} = U[(\alpha +1)I_n-\Lambda ]U^T{\mathscr {X}} \end{aligned}$$

8 $$\begin{aligned} {\mathscr {X}}_{hi}= & {} ({\mathscr {F}}_{hi}\star {\mathscr {X}})_{{\mathscr {G}}} = U[(\alpha -1)I_n+\Lambda ]U^T{\mathscr {X}} \end{aligned}$$

where $U=\{u_1,u_2,\ldots ,u_n\}$ is a set of orthogonal eigenvectors, while $\Lambda =diag([\lambda _1,\lambda _2,\ldots ,\lambda _n])$ is the corresponding eigenvalue. These are derived from ${\mathscr {A}}$’s standard Laplacian matrix, i.e., $L=I_n-D^{-1/2}AD^{-1/2}=U\Lambda U^T$. Thus, we can obtain the ${\mathscr {X}}_{lo}^{i}$ and the ${\mathscr {X}}_{hi}^{i}$ in $x_i$, i.e., the *i*-th node representation.

#### Node representation aggregation combining high- and low-frequency information

**Figure 1:**
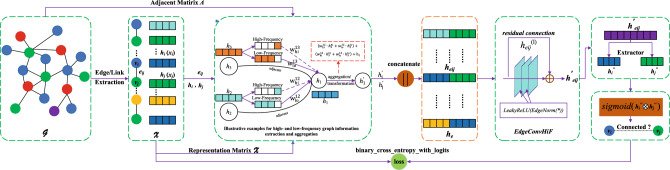
A GNN framework based on edge convolution, designed for link prediction in complex networks and named *EdgeConvHiF*, combines both high- and low-frequency information. It should be noted that this article only uses the representation aggregation and transformation process of node $v_1$ to illustrate how to fuse the high- and low-frequency graph information of nodes for node representation learning, and this process corresponds to Eq. 11, as illustrated in red box. Other nodes follow the same aggregation and transformation process.

Different from the traditional graph neural network node representation aggregation scheme that directly aggregates neighbor nodes, the aggregation of node representations that fuse high- and low-frequency information needs to consider the respective proportion of high- and low-frequency information in neighbor node representations. Intuitively, the attention mechanism that can adaptively perceive the weight of high- and low-frequency information in neighbor representation is employed in this paper. The weight $w_{lo}^{ij}$ and $w_{hi}^{ij}$ for nodes aggregating are shown in Eqs. 9 and 10, respectively.

9 $$\begin{aligned} w_{lo}^{ij}= & {} softmax_{j}({\mathscr {X}}_{lo}^{e_{ij}})=\frac{exp({\mathscr {X}}_{lo}^{e_{ij}})}{\sum _{k\in {\mathscr {N}}(i)}exp({\mathscr {X}}_{lo}^{e_{ik}})} \end{aligned}$$

10 $$\begin{aligned} w_{hi}^{ij}= & {} softmax_{j}({\mathscr {X}}_{hi}^{e_{ij}})=\frac{exp({\mathscr {X}}_{hi}^{e_{ij}})}{\sum _{k\in {\mathscr {N}}(i)}exp({\mathscr {X}}_{hi}^{e_{ik}})} \end{aligned}$$

thus, combing the situation of Eqs. 2 and 3, the representation $h_{i}^{(l)}$ of node $v_i$ can be manipulated by Eq. 11. And the intuitive and visual description of this process can refer to the illustrative example in Fig. 1.

11 $$\begin{aligned} h_{i}^{(l)}= w_{lo}^{ij}(({\mathscr {F}}_{lo}\star {\mathscr {X}})_{{\mathscr {G}}})_i + w_{hi}^{ij}(({\mathscr {F}}_{hi}\star {\mathscr {X}})_{{\mathscr {G}}})_i + h_{i}^{(l-1)} \end{aligned}$$

Accordingly, we further obtain the representation of edge $e_{ij}$ through Eqs. 3 and 4, as shown in Eq. 12.

12 $$\begin{aligned} \begin{aligned} h_{e_{ij}}^{(l)}=&(( w_{lo}^{im}(({\mathscr {F}}_{lo}\star {\mathscr {X}})_{{\mathscr {G}}})_i + w_{hi}^{im}(({\mathscr {F}}_{hi}\star {\mathscr {X}})_{{\mathscr {G}}})_i+h_{i}^{(l-1)})~|| \\&( w_{lo}^{jk}(({\mathscr {F}}_{lo}\star {\mathscr {X}})_{{\mathscr {G}}})_j + w_{hi}^{jk}(({\mathscr {F}}_{hi}\star {\mathscr {X}})_{{\mathscr {G}}})_j+h_{j}^{(l-1)})) \end{aligned} \end{aligned}$$

### Link representation normalization

We further investigate the impact of transformation manipulations in GNN layers on the performance of link prediction models. As highlighted by Zhou et al.^24^ and Zhang et al.^3^, the performance of GNNs deteriorates and experiences rapid fluctuations with increasing network depth, suggesting a growing problem of training instability. Current regularization methods, such as Dropout, as mentioned in Zhou et al.^24^, cannot effectively address these issues. Drawing inspiration from Zhou et al.^24^ and our previous work^3^, we propose an edge representation normalization technique named *EdgeNorm*. This method employs its own mean $\mu _{e_{ij}}^{(l)}$ and standard deviation $\sigma _{e_{ij}}^{(l)}$, as demonstrated in Eqs. 13 and 14, respectively.

13 $$\begin{aligned} \mu _{e_{ij}}^{(l)}= & {} \frac{1}{d_l}\sum _{k=0}^{d_l-1}{h_{e_{ij}k}^{(l)}} \end{aligned}$$

14 $$\begin{aligned} \sigma _{e_{ij}}^{(l)}= & {} \sqrt{\frac{1}{d_l}\sum _{k=0}^{d_l-1}{(h_{e_{ij}k}^{(l)}-\mu _{e_{ij}}^{(l)})}} \end{aligned}$$

where $d_l$ is the edge representation vector dimension; therefore, the strategy *EdgeNorm* is given in Eq. 15.

15 $$\begin{aligned} EdgeNorm(h_{e_{ij}}^{(l)})=\frac{h_{e_{ij}}^{(l)}-\mu _{e_{ij}}^{(l)}}{\sigma _{e_{ij}}^{(l)}} \end{aligned}$$

where $\mu _{e_{ij}}^{(l)}$ and $\sigma _{e_{ij}}^{(l)}$ represent the elementwise mean and deviation of edge $e_{ij}$ demonstrated in Eqs. 13 and 14, respectively. Consequently, an *EdgeConvHiF* layer, combined with *EdgeNorm* and a residual connection, results in Eq. 16.

16 $$\begin{aligned} h_{e_{ij}}^{(l+1)}=LeakyReLU(EdgeNorm(h_{e_{ij}}^{(l)}))+ h_{e_{ij}}^{(l)} \end{aligned}$$

Thankfully, incorporating the *EdgeConvHiF* layer into a widely-used deep graph learning framework is straightforward (In this paper, we utilize pytorch_geometric to implement *EdgeConvHiF* for link prediction within complex networks). Components like *binary_cross_entropy_with_logits* are employed to estimate the model performance.

### Link prediction framework

In this section, we outline the construction of a comprehensive framework for link prediction in complex networks, which incorporates the *EdgeConvHiF* and a binary classifier called *sigmoid*, as depicted in Fig. 1. We start by developing the *EdgeConvHiF* for link representation learning by stacking the edge convolutional layers, as indicated in Eq. 16. Following this, we employ a binary classifier, *sigmoid*, on the Hadamard product of two node representations acquired from the learned link representation through Eq. 17, resulting in a link predictor as expressed in Eq. 18. Lastly, to improve and optimize the *EdgeConvHiF*’s performance, we apply the *binary_cross_entropy_with_logits* loss function from the *pytorch_geometric*. The complete procedure described above can be observed in Fig. 1.

17 $$\begin{aligned} h_i,h_j= & {} Extractor(h_{e_{ij}}) \end{aligned}$$

18 $$\begin{aligned} f(v_i,v_j)= & {} sigmod(h_i\otimes h_j) \end{aligned}$$

where *Extractor* can extract the representations of $v_i$ and $v_j$ from the $e_{ij}$, while $\otimes$ denotes the Hadamard product manipulation. Moreover, $f(v_i,v_j)$ is a binary classifier *sigmoid*, which can decide whether there is a connection between $v_i$ and $v_j$.

## Experiments and discussion

To thoroughly assess the performance of * EdgeConvHiF*, we adhere to the experimental framework outlined in our prior research^3^. We conduct extensive experiments across various datasets and compare the results to different baseline methods. We first present the experimental settings, benchmark datasets, baseline techniques, and performance evaluation metrics relevant to the experiment. Subsequently, we examine the experimental outcomes to scrutinize the stability and reliability of *EdgeConvHiF*.

### Settings

The experimental settings in this article are as follows. Workstation Server: Dell T640, Operating System: CentOS-7-x86_64-DVD-1611, GPU: Tesla V100s, CUDA: 10.2, Python 3.7, PyTorch 1.11, and torch_geometric 2.1.

### Datasets

Three distinct and popular benchmark datasets are utilized as described by Zhang et al.^3^, namely Cora, CiteSeer, and PubMed^25–27^. These datasets pertain to academic citation networks where nodes symbolize documents and edges signify citation relationships. Besides, each document is associated with a label and possesses a specific set of features. Without loss of generality, we employ a ratio of 0.7, 0.2 and 0.1 to split each benchmark dataset for model training, validation and testing, respectively. The fundamental statistics for these datasets can be found in Table 2.

**Table 2:** Benchmark dataset statistics.

Dataset	#Nodes	#Links	#Classes	#Features
Cora	2708	5429	7	1433
CiteSeer	3327	4732	6	3703
PubMed	19,717	44,338	3	500

In accordance with the experimental configurations described in^25–27^, we solely consider the largest connected components for our experiments.

### Baselines

We assess the performance of *EdgeConvHiF* by comparing it to cutting-edge GNNs, such as GCN^19^, GAT^28^, EdgeConv^29^ and EdgeConvNorm^3^. It is worth noting that the GNNs used are solely for learning network node representations, and link prediction in complex networks can only be accomplished after adding the same *sigmoid *classifier employed for EdgeConvHiF. A concise overview of these GNNs is provided below.

- **GCN**^19^. As one of the most representative GNNs, the GCN’s core idea is that the central node learns its new representation by ‘hard’ aggregating the representations of its neighbors without considering the differences between nodes. While GCN has demonstrated outstanding performance in a variety of graph mining tasks, including node classification and link prediction.
- **GAT**^28^. Fortunately, compared with the GCN, GAT employs a ’soft’ aggregation approach for neighbor node representations in order to learn the central node representation, meaning that each neighboring node is assigned a weight based on its importance. Therefore, GAT can be regarded as a variant of GCN.
- **EdgeConv**^29^. What makes *EdgeConv* unique, compared to the GCN and its variants, is that it learns the representation of edge-associated nodes at the same time and has a better performance in the field of dense point cloud data.
- **EdgeConvNorm**^3^. To enable *EdgeConv* to achieve better performance in sparse and complex networks, *EdgeConvNorm* improves the edge convolution strategy, introduces the edge representation normalization strategy and obtains better link prediction performance than *EdgConv*. However, since the high-frequency representation information is not taken into account, there is still much room for improvement in its link prediction performance.

### Evaluation indicator

The AUC is a widely recognized metric for evaluating the performance of link prediction models. It is employed by various traditional models like *Jaccard*^30^ and *HPI*^31^, as well as GNN-based methods such as *EdgeConvNorm*^3^ and *SEAL*^32^. It is important to highlight that the AUC demonstrates the balance between the true positive rate ($TPR=TP/(TP+FN)$) and the false positive rate ($FPR=FP/(FP+TN)$). Thankfully, the AUPR (Area Under the Precision-Recall Curve) acts as a complement to AUC. This is particularly relevant because AUC might not be optimal when there is a significant imbalance between the positive and negative classes, and AUPR adjusts for this issue. Moreover, AUPR is valuable when our focus leans more toward the positive class over the negative class. The PR curve illustrates precision ($TP/(TP+FP)$) against recall ($FP/(TP+FN)$), and therefore, AUPR represents the area under this PR curve. In this research, the evaluation of performance for *EdgeConvHiF* relies on AUC and AUPR as metrics, while other metrics will be explored in future investigations. We run each model 10 times and present the mean value and associated standard error of AUC and AUPR as the final results, as demonstrated in Eqs. 19 and 20 for AUC, respectively. And AUPR follows the same strategy as mentioned above.

19 $$\begin{aligned} {\overline{AUC}}= & {} \frac{1}{n}\sum _{i=1}^{n}{AUC_i} \end{aligned}$$

20 $$\begin{aligned} \sigma _{AUC}= & {} \frac{\sqrt{\sum _{i=1}^{n}(AUC_i-{\overline{AUC}})^2}}{n} \end{aligned}$$

where $n=10$, and $AUC_i$ indicates the best result of the *i*-th run of the corresponding model listed in section “Baselines”.

### Experimental results and discussion

**Figure 2:**
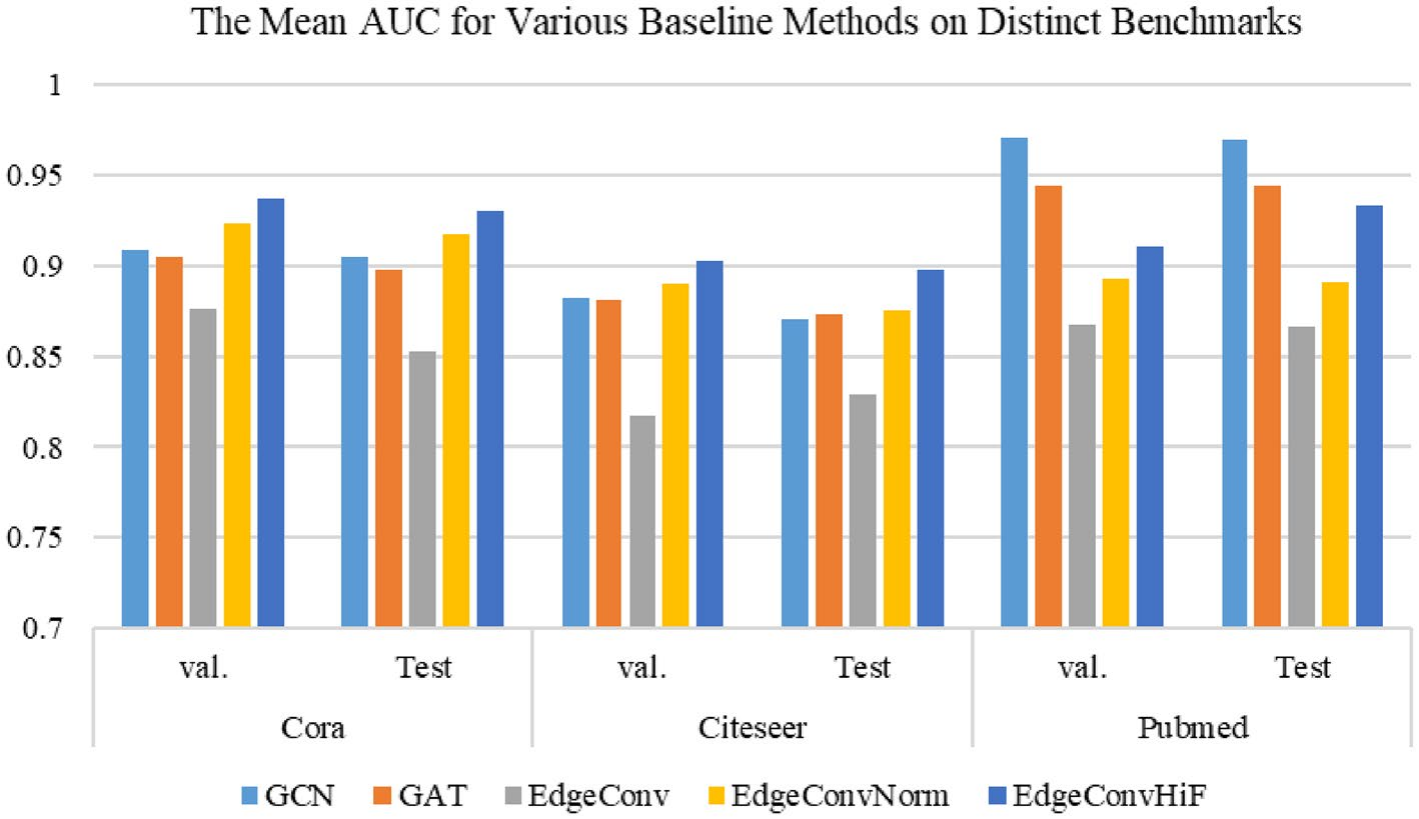
The mean AUC obtained from various baseline methods on distinct benchmarks, where *val*. represents the validation dataset and *Test* indicates the dataset for test.

**Figure 3:**
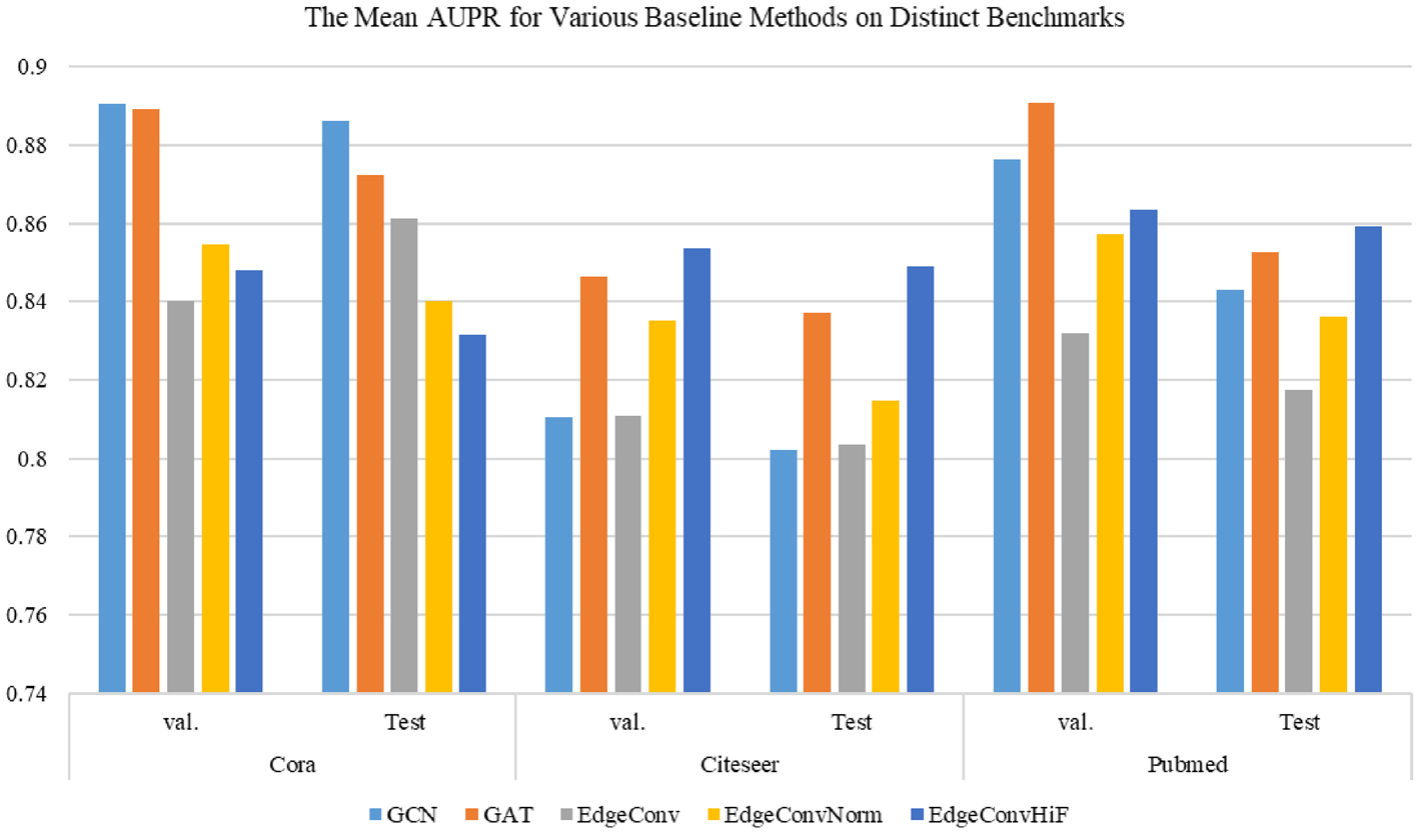
The mean AUPR obtained from various baseline methods on distinct benchmarks, where *val*. represents the validation dataset and *Test* indicates the dataset for test.

The experimental configurations are described as follows: a learning rate of 0.001, 256 hidden channels, 256 output channels, 5000 epochs, and 10 runs. The models, including *GCN*, *GAT*, *EdgeConv*, *EdgeConvNorm* and *EdgeConvHiF*, are implemented using $torch\_geometric$ in an identical hardware and software environment. Additionally, a *Dropout* layer is incorporated into each model, with a probability *p* of 0.6 for both Cora and CiteSeer, and 0.7 for PubMed. The amount of heads for GAT is set to 1. The best comparative experimental results for all models are presented in Tables 3 and 4. Moreover, for the purpose of better assisting readers in observing and understanding the experimental results, the graphical representations corresponding to the experimental results in Tables 3 and 4 are shown in Figures 2 and 3, respectively.

**Table 3:** The experimental outcomes are obtained from various baseline methods on distinct benchmarks, based on the metric of AUC.

Dataset		Model				
		GCN	GAT	EdgeConv	EdgeConvNorm	EdgeConvHiF
Cora	Val.	0.9089 ± 0.0022	0.9043 ± 0.0047	0.8759 ± 0.0069	0.9231 ± 0.0119	**0.9371** ± **0.0028**
	Test	0.9050 ± 0.0026	0.8979 ± 0.0019	0.8528 ± 0.0082	0.9178 ± 0.0088	**0.9298** ± **0.0016**
Citeseer	Val.	0.8816 ± 0.0045	0.8808 ± 0.0052	0.8174 ± 0.0060	0.8896 ± 0.0080	**0.9027** ± **0.0008**
	Test	0.8701 ± 0.0039	0.8731 ± 0.0037	0.8294 ± 0.0084	0.8754 ± 0.0066	**0.8978** ± **0.0018**
Pubmed	Val.	**0.9708** ± 0.0006	0.9436 ± 0.0012	0.8675 ± 0.0026	0.8930 ± 0.0066	0.9104 ± 0.0026
	Test	0.**9694** ± **0.0004**	0.9436 ± 0.0006	0.8665 ± 0.0018	0.8911 ± 0.0025	0.9328 ± 0.0013

It is important to mention that we reference the results from Reference^3^ for all models, with the exception of *EdgeConvHiF*. Significant values are in [bold].

**Table 4:** The experimental consequences are obtained from various baseline methods on distinct benchmarks, based on the metric of AUPR.

Dataset		Model				
		GCN	GAT	EdgeConv	EdgeConvNorm	EdgeConvHiF
Cora	Val.	**0.8905** ±**0.0102**	0.8893 ± 0.0016	0.8402 ± 0.0117	0.8547 ± 0.0079	0.8479 ± 0.0012
	Test	**0.8863** ± **0.0106**	0.8725 ± 0.0027	0.8613 ± 0.0049	0.8401 ± 0.0104	0.8315 ± 0.0027
Citeseer	Val.	0.8103 ± 0.0106	0.8464 ± 0.0031	0.8107 ± 0.0046	0.8352 ± 0.0117	**0.8537** ± **0.0015**
	Test	0.8021 ± 0.0026	0.8371 ± 0.0025	0.8035 ± 0.0109	0.8147 ± 0.0037	**0.8491** ± ** 0.0038**
Pubmed	Val.	0.8764 ± 0.0043	**0.8907** ± **0.0039**	0.8320 ± 0.0019	0.8571 ± 0.0021	0.8635 ± 0.0048
	Test	0.8429 ± 0.0037	0.8526 ± 0.0074	0.8173 ± 0.0047	0.8362 ± 0.0011	**0.8593** ± **0.0071**

Significant values are in [bold].

From the information presented in Tables 3 and 4, as well as the visual representations in Figs. 2 and 3, we can make the subsequent observations.

**Figure 4:**
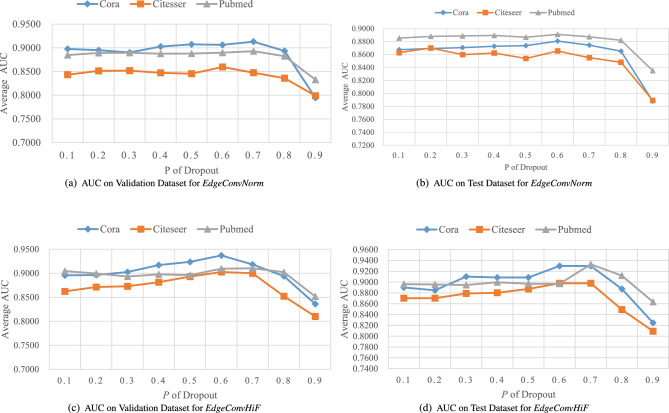
The mean AUC of *EdgeConvHiF* tested on different datasets with different Dropout probabilities. It is important to note that both (**a**) and (**b**) are directly derived from the findings in^3^.

1. In this paper, the proposed *EdgeConvHiF* model surpasses its predecessors, *EdgeConv* and *EdgeConvNorm*, in performance on benchmark datasets such as Cora, CiteSeer, and PubMed. This improvement is attributed to the edge convolutional manipulations that merge high- and low-frequency graph information. Taking into account the findings from reference^3^ and the varied performance of different *Dropout* probabilities depicted in Fig. 4, it can be stated that incorporating high- and low-frequency information in edge convolution along with the normalization strategy results in a more stable link prediction model performance. This, in turn, enhances the learning and smoothing of link representations.
2. Although the performance of *EdgeConvHiF* is not significantly improved compared with these of the classic GCN and even GAT, as a benchmark, it has higher performance and stronger stability than its predecessors, *EdgeConv* and *EdgeConvNorm*, especially on Cora and CiteSeer. In addition, the performance of *EdgeConvHiF* is only slightly lower than that of GCN. The main explanations for the aforementioned issues are summarized as follows.
  Large-scale networks, such as PubMed, typically exhibit lower community modularity and network density, as outlined in Reference^3^. The network density of PubMed is 0.00023, which is considerably lower than that of Cora, which has a network density of 0.00148. Even though the *EdgeConvHiF* method effectively integrates both low-frequency and high-frequency information from neighboring node representations using specific weights for learning link representations, it struggles to fully and efficiently learn the corresponding link representations due to PubMed’s high sparsity and scale-free nature.
  Moreover, as indicated in Table 2, PubMed has only 500 features, in contrast to Cora and CiteSeer. Furthermore, while PubMed is substantially larger in size compared to both Cora and CiteSeer, the limited number of features hinders the *EdgeConvHiF* method’s ability to effectively learn link representations from the PubMed dataset.
  Additionally, the low AUPR values presented in Table 4 once again confirm the sparsity of PubMed and the imbalance between positive and negative classes. It is worth noting that although the AUPR values presented in Table 4 are generally lower than the corresponding AUC values in Table 3, the test results of different baselines on different datasets have little fluctuation and are relatively stable, thus once again demonstrating the stability of the *EdgeConvHiF* proposed in this article.
3. Thankfully, the performance of *EdgeConvHiF* is stable, the AUC values are all above 89% and the AUPR values are all above 85%, and no any instability phenomenon occurs. Although the GCN and GAT have good link prediction performance on medium-scale networks, they are more suitable for large-scale networks such as PubMed. However, *EdgeConvHiF* performs well in networks with different scales and features.

### Model stability

To our knowledge, *Dropout* can randomly deactivate certain neurons within the graph neural network during the *EdgeConvHiF* training process. Naturally, the associated weights will not be updated during this time, but they will be temporarily stored and used for subsequent training. This approach enhances the model’s generalization capabilities while mitigating the issue of overfitting.

**Table 5:** An AUC comparison was carried out on both validation and test datasets with varying Dropout probabilities.

Dataset	Cora		CiteSeer		PubMed	
P	Val.	Test	Val.	Test	Val.	Test
0.1	0.8956 ± 0.0032	0.8901 ± 0.0103	0.8623 ± 0.0127	0.8703 ± 0.0124	**0.9048**± **0.0005**	**0.8962** ± **0.0016**
0.2	0.8964 ± 0.0003	0.8847± 0.0015	0.8714 ± 0.0089	0.8698 ± 0.0071	**0.8996**± **0.0011**	**0.8956** ± **0.0024**
0.3	**0.9025** ± **0.0073**	**0.9101** ± **0.0031**	0.8729 ± 0.0074	0.8788 ± 0.0042	0.8932± 0.0023	0.8947 ± 0.0006
0.4	**0.9171**± **0.0059**	**0.9084**± **0.0012**	0.8814±0.0015	0.8803±0.0027	0.8977±0.0006	0.8995±0.0015
0.5	**0.9238**± **0.0038**	**0.9192**± **0.0037**	0.8931±0.0131	0.8874±0.0081	0.8962±0.0033	0.8968±0.0012
0.6	**0.9371**± **0.0028**	**0.9298**± **0.0016**	0.9027±0.0008	0.8978±0.0018	0.9097±0.0021	0.8994±0.0070
0.7	**0.9184**± **0.0107**	0.8103±0.0107	0.9001±0.0094	0.8852±0.0047	0.9104±0.0026	**0.9328**± **0.0013**
0.8	0.8941±0.0123	0.8874±0.0102	0.8524±0.0122	0.8493±0.0055	**0.9024**±**0.0003**	**0.9120**±**0.0032**
0.9	0.8362±0.0079	0.8247±0.0139	0.8101±0.0161	0.8092±0.0093	**0.8518**±**0.0027**	**0.8633**±**0.0012**

The terms Val. and Test refer to the validation and test dataset, respectively. Significant values are in [bold].

To further investigate the stability of *EdgeConvHiF*, we adopted the same scenario described in References^3,33^, wherein the probability *p* of *Dropout* varies from 0.1 to 0.9 in increments of 0.1. As AUPR is a supplement to AUC, this article only uses the metric of AUC to evaluate the stability of the model *EdgeConvHiF*. Subsequently, the corresponding AUCs were analyzed to assess the stability of *EdgeConvHiF*. As seen in Fig. 4c,d and Table 5, excluding the case of $p=0.9$, the mean AUC of different baselines for the three benchmark datasets in Table 2 changes gradually, with a gap of nearly 0.04. This indicates that under the influence of varying neuron dropout rates, changes in *p* have minimal impact on *EdgeConvHiF*’s performance, demonstrating the model’s stability and robustness.

Additionally, by examining the experimental results of *EdgeConvHiF* presented in Fig. 4a,b and the mean AUC with standard error shown in Table 5, the model achieves an AUC greater than 0.89, which is 0.03 higher than that of *EdgeConvNorm*, with small standard errors. The AUC change trend of *EdgeConvHiF* is smoother compared to *EdgeConvNorm*.

However, in the case of $p=0.9$, both the average AUC and the corresponding standard error are reduced. This can be mainly attributed to the increased value of *p* resulting in fewer neurons being retained in *EdgeConvHiF* for learning link representations, leading to suboptimal link prediction performance and unsatisfactory outcomes.

## Conclusions

In this study, we introduce a link prediction framework called *EdgeConvHiF*, which is based on edge convolution and combines both high- and low-frequency information. Additionally, the framework incorporates a link representation normalization strategy to optimize *EdgeConvHiF*’s performance. The process begins with extracting high- and low-frequency information from node representations, followed by using an attention mechanism to merge this information for learning link representations. Following that, representations of nodes are obtained from the link representation, and a binary classifier, *sigmoid*, is employed on the Hadamard products of these representations to assess the existence of a link between nodes. Comprehensive experiments conducted on benchmarks demonstrate that *EdgeConvHiF* exhibits strong performance and holds benefits over current baseline approaches.

Nonetheless, there are at least two areas that warrant further enhancement. Firstly, although AUC and AUPR are widely-employed metric , as exemplified by those in^30,32^, it is crucial to investigate alternative metrics, such as accuracy and F-value. Secondly, to thoroughly assess *EdgeConvHiF*’s stability and applicability, it is necessary to test the method on large-scale, dynamic, and heterogeneous networks. Our future work will be dedicated to addressing these concerns and refining the approach accordingly.

## Data Availability

Data will be made available on request, and Z. Zhang can be contacted to obtain the data.
